# Many are called but few are chosen—from multiple clonal origins to one winner

**DOI:** 10.1093/lifemedi/lnaf008

**Published:** 2025-02-24

**Authors:** Qihang Chen, Zihan Liu, Bingjie Chen

**Affiliations:** GMU-GIBH Joint School of Life Sciences, The Guangdong-Hong Kong-Macao Joint Laboratory for Cell Fate Regulation and Diseases, Guangzhou Laboratory, Guangzhou Medical University, Guangzhou 510260, China; GMU-GIBH Joint School of Life Sciences, The Guangdong-Hong Kong-Macao Joint Laboratory for Cell Fate Regulation and Diseases, Guangzhou Laboratory, Guangzhou Medical University, Guangzhou 510260, China; GMU-GIBH Joint School of Life Sciences, The Guangdong-Hong Kong-Macao Joint Laboratory for Cell Fate Regulation and Diseases, Guangzhou Laboratory, Guangzhou Medical University, Guangzhou 510260, China; State Key Laboratory of Respiratory Disease, Department of Critical Care, Second Affiliated Hospital, Guangzhou Medical University, Guangzhou 510260, China

Understanding the origin and evolutionary dynamics of cellular changes at the onset of cancer is essential for effective early detection and intervention. While tumors were once thought to arise from a single progenitor cell [1], the debate between monoclonal and polyclonal origins persists [2–4]. Recent research by Lu et al. [2] has systematically demonstrated that polyclonal origins are prevalent. Using lineage tracing technology and single-cell transcriptome sequencing, the research team observed in mouse models and human precancerous lesion tissues that early tumor lesions frequently arise from multiple independent cellular clones. They also proposed a new model of “polyclonal-to-monoclonal transition” in tumorigenesis, providing a novel theoretical framework for the early evolution of tumors and offering innovative approaches for early cancer screening, risk prediction, and targeted early intervention.

Nearly 50 years ago, Cairns pointed out that each multicellular organism is compartmentalized into numerous small units, usually in the form of stem-cell niches. Each niche is an independently evolving entity, and occasionally, one might evolve into an out-of-control proliferative population, manifested clinically as a tumor. However, as an evolutionary unit, the population size (*N*) of each niche is estimated fewer than 50 in the human colon [5]. Given the existence of numerous small populations evolving in parallel, it is plausible that multiple clonal expansions could occur within an individual. Indeed, over the past 10 years, many studies have shown that normal tissues often consist of a large number of cellular clones of various sizes. Therefore, the architecture appears to predispose tissues to multiple origins of tumors.

**Figure 1.**
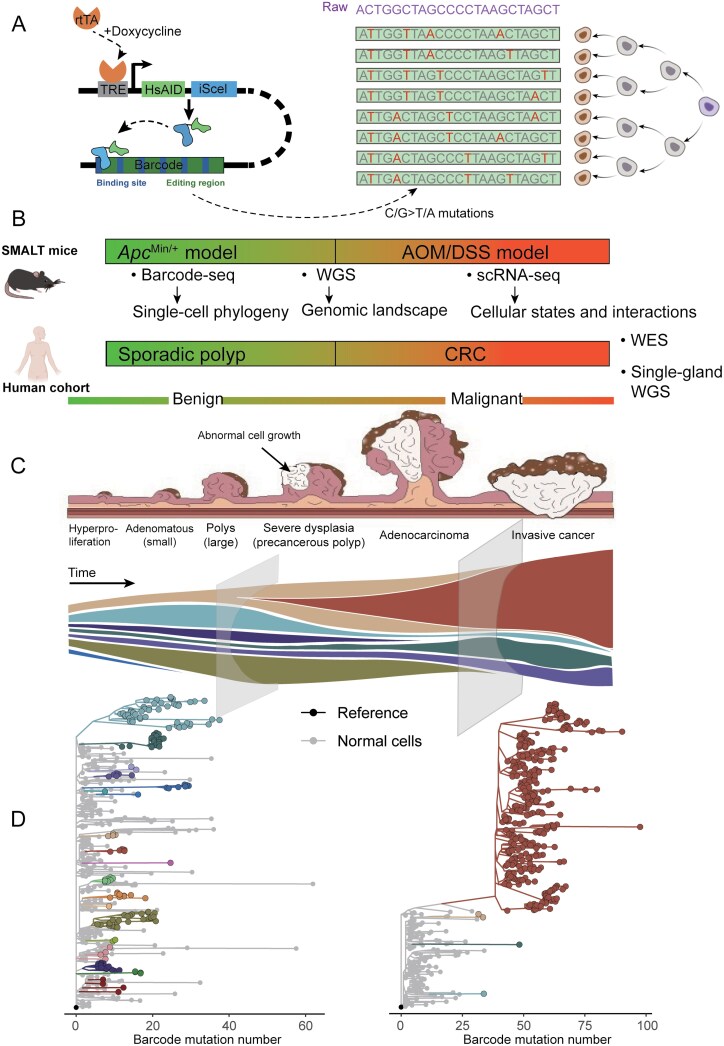
**Single-cell phylogenies and transcriptomes reveal a "polyclonal-to-monoclonal transition" model in colorectal cancer (CRC) development.** (A) Schematic representation of the SMALT lineage tracing system, illustrating how it enables the tracking of genetic lineages at the single-cell level, leading to the construction of phylogenetic trees that depict the evolutionary relationships among tumor cells. (B) Overview of the integrated models and data types utilized in the research by Lu et al., including the AOM/DSS and *Apc*^Min/+^ models, alongside DNA barcode mutations, whole-genome sequencing, and single-cell transcriptomics, which collectively inform the understanding of tumorigenesis. (C) Proposed "polyclonal-to-monoclonal transition" model illustrating the progression of colorectal cancer from benign hyperproliferation characterized by polyclonal origins to the emergence of monoclonal tumors, which are associated with increased malignancy and genomic alterations. (D) Visualization of single-cell phylogeny trees at both the polyclonal and monoclonal stages, highlighting the distinct evolutionary trajectories and clonal diversity present in early tumorigenesis, with a focus on the implications of these differences for tumor progression and therapeutic targeting.

To validate this hypothesis, extremely deep sampling of a limited number of tumors could reveal minor independent clones from somatic mutations [3]. However, given that the rate of somatic mutations is often inefficient, it would be challenging to conduct extensive studies across numerous cases. Furthermore, systematically studying the different stages of cancer development, such as polyps, benign, and malignant tumors, can also provide in-depth insights into the transition of clones during the process of tumorigenesis. In recent years, the development and application of new technologies such as lineage tracing have ushered in a new phase in tumor evolution research [6]. In Lu et al.'s study [2], the team has for the first time, harnessed a base editor-based lineage tracing technique, substitution mutation-aided lineage tracing (SMALT) [7], to systematically map the origin and evolution of intestinal precancers in mouse models at single-cell resolution (Fig. 1A).

Using transgenic mice equipped with the SMALT system, the research team established two models for studying colorectal cancer: the azoxymethane/dextran sulfate sodium (AOM/DSS) model for inflammation-induced cancer and the *Apc*^Min/+^ model for multiple polyposis (Fig. 1B). Their single-cell phylogenetic analysis revealed that the majority of inflammation-induced cancers and all Apc polyps originated from multiple clones. By combining DNA barcode mutations, whole-genome sequencing, and single-cell transcriptomics, they discovered that monoclonal tumors exhibit higher malignancy, suggesting that monoclonal tumors might signify a more advanced stage in tumor development. These insights led the team to propose a novel “polyclonal-to-monoclonal transition” model for tumorigenesis, offering a theoretical perspective on the early evolution of tumors.

The research team also assembled a cohort of 107 patients with sporadic colorectal polyps and concurrent colorectal cancer (Fig. 1B). Genomic sequencing data revealed that approximately 30% of human colorectal polyps originate from multiple clones and exhibit lower mutational burdens and copy number variations. Pathological characteristics also indicated that polyclonal polyps are smaller and less malignant compared to monoclonal polyps; in contrast, monoclonal polyps display greater genomic alterations [8, 9], larger sizes, and higher degrees of malignancy. These findings validate the lineage tracing results from mice and collectively support the model of early tumor evolution from polyclonal to monoclonal origins.

Based on these results, they proposed a new model for the polyclonal-to-monoclonal transition in early tumorigenesis (Fig. 1C and 1D), emphasizing the importance of specific genomic and microenvironmental changes, as well as cell–cell interactions in this process. These discoveries provide a novel conceptual framework for understanding cancer origins and suggest new strategies for early intervention by targeting cell–cell communication pathways. Consequently, this study paves the way for new research into the mechanisms of polyclonal formation and cell–cell interactions during early tumorigenesis. Recently, another study has also elaborated on the role of differential activation of oncogenic pathways between clones and their interactions in promoting tumorigenesis [10].

While the transition from polyclonal to monoclonal populations is clearly elucidated in this study, the underlying processes involved in the early stages of clonal evolution remain complex and warrant further investigation. A potential factor in the early origin and progression of tumors is “field cancellation.” The research team observed an increase in ligand–receptor interactions among epithelial subtypes in early polyclonal lesions, in contrast to both normal colon tissue and late monoclonal lesions. They further identified significantly enriched ligand–receptor interactions in early polyclonal lesions, primarily involving genes related to extracellular matrix (ECM) organization and cell adhesion. This suggests that extensive cell–cell interactions mediated by ECM organization and cell adhesion are characteristic of early inflammation-driven intestinal tumorigenesis. Understanding how these interactions promote tumor growth in the early stages will be crucial for the future prevention and diagnosis of cancer.

Furthermore, single-cell phylogenetic analyses by Lu et al. also revealed the presence of small clones coexisting with the dominant monoclonal populations in tumor samples. From an evolutionary perspective, while the early polyclonal stage of a tumor may appear cooperative, this cooperation is often unstable in the long term. Evolutionary pressures, including competition, mutations, and microenvironmental stress, ultimately shift the dynamics from cooperation to competition. In nature, different species exhibit varying adaptive advantages based on their reproductive strategies and resource utilization under different environmental conditions. Similarly, different clones in a tumor may adopt distinct growth strategies (such as r-selection or K-selection strategies) in response to changes in the microenvironment and resource availability.

In the early stages of tumor evolution, transient cooperation between clonal cells may occur as they work together to adapt to the tumor microenvironment. For example, some clones may specialize in angiogenesis (promoting tumor oxygenation and nutrient supply), while others focus on rapid proliferation, relying on the support provided by the former. This division of labor can enhance the overall growth capacity of the tumor, particularly in the early stages when resources are relatively abundant and the tumor is small. However, as the tumor progresses, mutations accumulate, leading to increased internal heterogeneity. Especially in high-density environments, some clones may acquire stronger growth advantages or immune evasion capabilities through genetic mutations, allowing them to dominate the tumor space. Consequently, other clones are gradually eliminated, and ultimately, monoclonal tumors replace polyclonal structures, potentially turning cooperation into competition. Therefore, the polyclonality and transition to monoclonality in tumors is a complex evolutionary process, awaiting further in-depth research.

The transition from polyclonal to monoclonal is significant for the early prevention and diagnosis of cancer. In the early stages of tumor development, cells originate from different clones, indicating lower malignancy. Over time, certain clones acquire mutational advantages, gradually dominating and leading to the formation of a monoclonal tumor. This transition is often accompanied by increased malignancy, enhanced drug resistance, and immune evasion. By detecting the clonal characteristics in tumors, we can differentiate between progression stages and identify high-risk types. Therefore, understanding and monitoring the “polyclonal-to-monoclonal transition” is crucial for early detection and personalized treatment strategies.
